# No Association of Cryptococcal Antigenemia with Poor Outcomes among Antiretroviral Therapy-Experienced HIV-Infected Patients in Addis Ababa, Ethiopia

**DOI:** 10.1371/journal.pone.0085698

**Published:** 2014-01-21

**Authors:** Christopher C. Smitson, Admasu Tenna, Mulugeta Tsegaye, Abere S. Alemu, Daniel Fekade, Abraham Aseffa, Henry M. Blumberg, Russell R. Kempker

**Affiliations:** 1 Department of Medicine, University of California, San Francisco School of Medicine, San Francisco, California, United States of America; 2 Division of Infectious Diseases, Department of Medicine, Addis Ababa University, Addis Ababa, Ethiopia; 3 All Africa Leprosy, TB and Rehabilitation Training Centre, Addis Ababa, Ethiopia; 4 Haramya University, Medical Laboratory Sciences, Harar, Ethiopia; 5 Armauer-Hansen Research Institute, Addis Ababa, Ethiopia; 6 Division of Infectious Diseases, Department of Medicine, Emory University School of Medicine, Atlanta, Georgia, United States of America; Imperial College London, United Kingdom

## Abstract

**Introduction:**

There are limited data on clinical outcomes of ART-experienced patients with cryptococcal antigenemia. We assessed clinical outcomes of a predominantly asymptomatic, ART-experienced cohort of HIV+ patients previously found to have a high (8.4%) prevalence of cryptococcal antigenemia.

**Methods:**

The study took place at All Africa Leprosy, Tuberculosis and Rehabilitative Training Centre and Black Lion Hospital HIV Clinics in Addis Ababa, Ethiopia. A retrospective study design was used to perform 12-month follow-up of 367 mostly asymptomatic HIV-infected patients (CD4<200 cells/µl) with high levels of antiretroviral therapy use (74%) who were previously screened for cryptococcal antigenemia. Medical chart abstraction was performed approximately one year after initial screening to obtain data on clinic visit history, ART use, CD4 count, opportunistic infections, and patient outcome. We evaluated the association of cryptococcal antigenemia and a composite poor outcome of death and loss to follow-up using logistic regression.

**Results:**

Overall, 323 (88%) patients were alive, 8 (2%) dead, and 36 (10%) lost to follow-up. Among the 31 patients with a positive cryptococcal antigen test (titers ≥1∶8) at baseline, 28 were alive (all titers ≤1∶512), 1 dead and 2 lost to follow-up (titers ≥1∶1024). In multivariate analysis, cryptococcal antigenemia was not predictive of a poor outcome (aOR = 1.3, 95% CI 0.3–4.8). A baseline CD4 count <100 cells/µl was associated with an increased risk of a poor outcome (aOR 3.0, 95% CI 1.4–6.7) while an increasing CD4 count (aOR 0.1, 95% CI 0.1–0.3) and receiving antiretroviral therapy at last follow-up visit (aOR 0.1, 95% CI 0.02–0.2) were associated with a reduced risk of a poor outcome.

**Conclusions:**

Unlike prior ART-naïve cohorts, we found that among persons receiving ART and with CD4 counts <200 cells/µl, asymptomatic cryptococcal antigenemia was not predictive of a poor outcome.

## Introduction

Recent reports highlight the alarming issue of cryptococcal meningitis in Sub-Saharan Africa and make it clear that there is still much to be done to improve the diagnosis, management, and prevention of cryptococcal disease [1], [2]. In response to the high burden of cryptococcal disease in resource limited settings (RLS) including an estimated 720,000 cases of cryptococcal meningitis (CM) and 530,000 deaths annually in Sub-Saharan Africa [1], World Health Organization (WHO) recently released “rapid advice” guidelines for cryptococcal infection among persons living with HIV [3]. A major emphasis of the guidelines is to consider implementation of cryptococcal antigen (CRAG) screening among antiretroviral therapy (ART)-naïve adults with a CD4 count <100 cell/µl in areas with a high prevalence of cryptococcal infection, followed by preemptive anti-fungal therapy for those with a positive CRAG test [3]. However, this conditional recommendation was based on low quality evidence and more data are needed to optimize cryptococcal screening strategies.

Previous CRAG screening studies included only ART-naïve individuals, and there are no data on the clinical benefit of screening ART-experienced HIV patients. Accordingly, WHO guidelines for CRAG screening do not include recommendations for individuals who are already receiving ART [3]. In a prior cross sectional study of 367 ART-experienced HIV-infected individuals with a CD4<200 cells/µl in Addis Ababa, Ethiopia, we found a serum cryptococcal antigenemia prevalence of 8.5% [4]. The purpose of this current study was to assess one-year clinical outcomes for this cohort in an effort to determine the utility of CRAG screening among an ART-experienced cohort.

## Methods

### Study Design and Patients

A retrospective study design was utilized to perform a 12-month clinical follow-up of 367 HIV-infected patients from our prior CRAG screening study. The design and results of the baseline screening assessment, performed at two ART clinics in Addis Ababa, Ethiopia, have been reported previously [4].

### Follow-up

Patient follow-up was performed from June-August 2012, approximately one year after enrollment in the baseline study. The following information was gathered by medical chart and computer registry data abstraction: clinic visit history, ART use, CD4 counts, development and treatment of opportunistic infections including cryptococcal disease, lumbar puncture results (if performed) and whether the patient was alive, dead, or lost to follow-up. For the deceased, cause of death was ascertained from the medical chart. Loss to follow-up was defined as no follow-up visits or not having visited the clinic within the last 6 months. For those who were lost to follow-up and/or medical record could not be found, patient information was cross-referenced with a computer registry at both sites. Registry data included dates of past clinic visits, CD4 count results and whether the patient had died. Written informed consent was obtained from all participants for the baseline CRAG screening. Study approval was obtained from the Institutional Review Boards of Emory University, Addis Ababa University, and the Armauer Hansen Research Institute.

### Data Management

All data were entered into an online REDCap database [5], and data analyses were performed using SAS software (v.9.3). The primary study result was a poor outcome, which was defined as a composite of death and lost to follow-up. For comparing characteristics among patients alive versus those with a poor outcome, differences in categorical variables were tested using a χ^2^ statistic, and for continuous variables a two-sample *t*-test was performed. Univariate and multivariate logistic regression were performed to evaluate the association of cryptococcal antigenemia as well as other potential confounding risk factors and a poor outcome. Risk factors with biologic plausibility or known to be associated with HIV clinical outcomes were included in the multivariate model. Model building and selection was based on the purposeful selection of covariates strategy as previously described [6]. A p value <0.05 was considered statistically significant.

## Results and Discussion

### Clinical Follow-up

**Figure 1:**
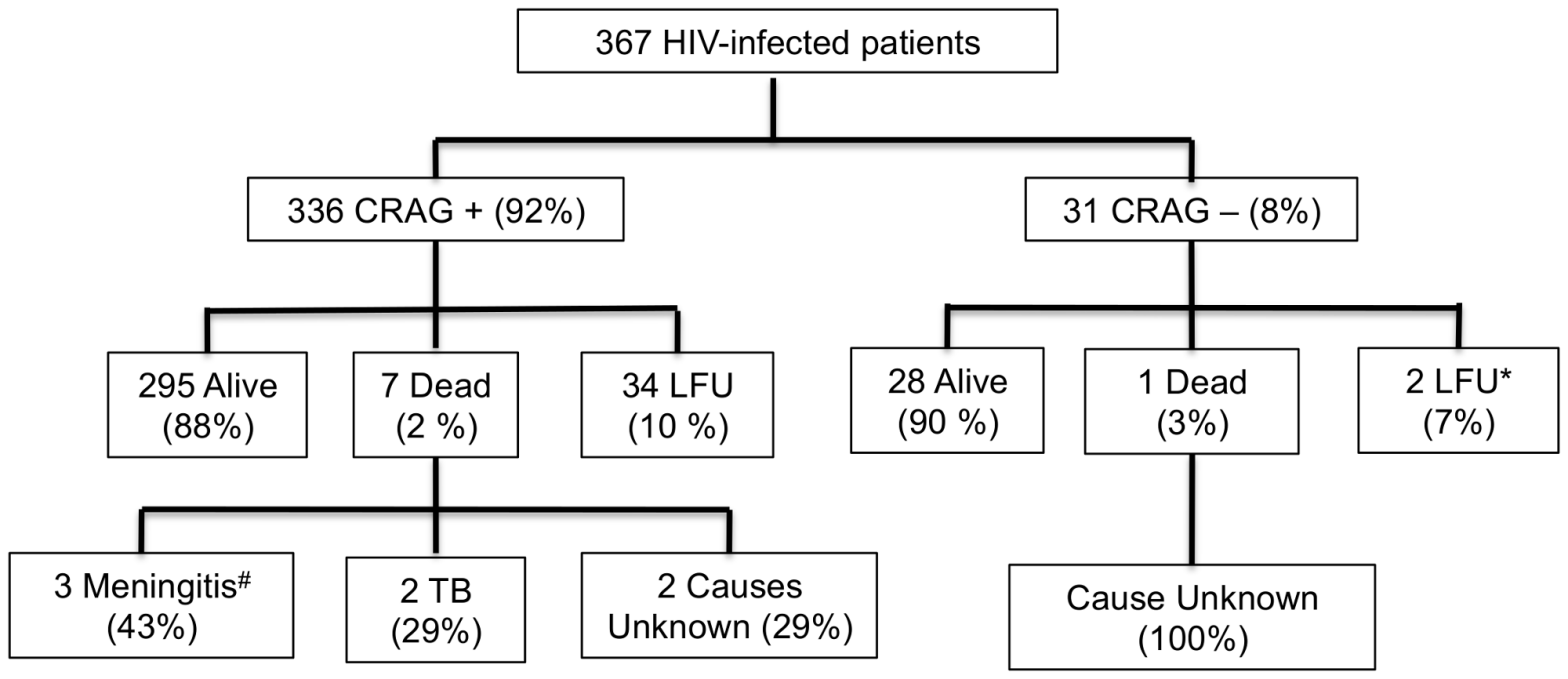
One-Year Follow Up of 367 HIV-Infected Patients, Addis Ababa, Ethiopia. Legend: *No clinic Follow-Up in ≥6 months, 16 patients with no follow up at all; ^#^1 received treatment for CM; CRAG, serum cryptococcal antigen; CM, cryptococcal meningitis; LFU, loss to follow-up.

A total of 367 HIV-infected patients had one-year follow-up performed including 31 persons with baseline cryptococcal antigenemia. None of the patients were clinically suspected to have meningitis at baseline. In regards to clinical outcomes at time of follow-up, 323 persons were alive (88%), 8 were confirmed dead (2%) and 36 were lost to follow-up (10%) (Figure 1). Of patients lost to follow-up, 16 did not have any follow-up visits and 20 patients had at least one follow-up visit.

Among the 31 patients with cryptococcal antigenemia, 28 were alive (90%), 1 dead (3%), and 2 lost to follow-up (7%) (Figure 1). Twenty-six (84%) of 31 were receiving ART at baseline and all 28 individuals who were alive at follow-up were on ART at follow-up. All three patients with a CRAG titer of ≥1∶1024 were either dead (1) or lost to follow-up (2). The patient who died had a baseline CD4 count of 45 cells/µl (n = 1) with no follow-up measurement, never received ART or anti-fungal therapy, and died of an unknown cause. Both patients lost to follow-up were receiving ART and had follow-up CD4 counts (5 and 13 cells/µl). One of the patients lost to follow-up was hospitalized three times for meningitis of unknown etiology and received fluconazole treatment empirically for CM each time. No persons with cryptococcal antigenemia received a lumbar puncture at baseline. A total of three patients (2 alive, 1 lost to follow-up) received fluconazole during follow-up.

Among patients with a negative CRAG test at baseline, 295 of 336 were alive (88%), 7 dead (2%) and 34 were lost to follow-up (10%) (Figure 1). Among the 7 patients with a negative CRAG test who died, 2 died of meningitis of unknown etiology, 1 of CM, 2 of tuberculosis, and 3 of unknown etiology (Figure 1).

### Risk Factors for Composite Outcome of Death or Lost to Follow-up

In univariate analysis, patients with a CD4 count <100 cells/µl at baseline were more likely to have a poor outcome compared to those patients with a CD4 count of 100–200 cells/µl (OR = 2.97, 95% CI 1.56–5.63). ART use at baseline and last follow-up visit and an increasing CD4 count were associated with a reduced risk of a poor outcome (Table 1).

**Table 1:** Comparison of Characteristics in Those who are Alive vs. Those who Died/Lost to Follow up.

Characteristic	Total (%)	Alive (%)	Death/LFU (%)	P*
	n = 367	n = 323	n = 44	
Cryptococcal Antigen Positive	31 (9)	28 (9)	3 (7)	0.68
Demographics				
Mean Age in years (IQR)	36.7 (30–41)	36.9	35.1	0.20
Male	163 (45)	141 (44)	22 (50)	0.44
BMI <18.5 kg/m^2^	97 (26)	81 (25)	16 (36)	0.11
Site 1 vs. 2	262 (71)	229 (71)	33 (75)	0.57
ART Use				
ART at Baseline	270 (74)	245 (76)	25 (57)	0.006
ART at Follow-Up (n = 351)	328 (94)	314 (98)	14 (48)	<.0001
Median time on ART in patients on ART at Follow-Up^#^ (months) [IQR]	30.5 (13–64)	32 (14–66)	7.7 (5–13)	<.01
CD4 Count Status				
CD4<100 vs. 100–200 at Baseline	117 (32)	93 (29)	24 (55)	0.001
Mean CD4 Increase at Follow-Up (n = 351) [IQR]	75 (1–122)	79.5	36.1	0.02
Opportunistic Infections				
Treated for TB at Baseline or during follow-up	43 (12)	38 (12)	5 (11)	0.94
Treated for Pneumonia at baseline or during follow-up	42 (11)	37 (12)	5 (11)	0.99
Herpes Zoster at baseline or during follow-up	35 (10)	30 (9)	5 (11)	0.66

CRAG, serum cryptococcal antigen; BMI, body mass index; ART, antiretroviral therapy; OI, opportunistic infection; TB, tuberculosis; URI, upper respiratory infection acid. Other risk factors with not enough statistical numbers to analyze: alcohol use (11), Illicit Drug Use (7), Diabetes Mellitus (1), Hepatitis (3), Malignancy (8). *Univariate analysis; ^#^Missing data for 22 patients.

In multivariate analysis, there was no association between serum cryptococcal antigenemia and a poor outcome (aOR = 1.3, 95% CI 0.3–4.8) (Table 2). Independent risk factors for a poor outcome included a CD4 count <100 cells/µl at baseline (aOR 3.0, 95% CI 1.4–6.7); any increase in CD4 count (aOR 0.12, 95% CI 0.05–0.27) and receiving ART at last follow-up visit (aOR 0.06, 95% CI 0.02–0.19) (Table 2).

**Table 2:** Univariate and Multivariate Analysis of Risk Factors for Poor Outcomes Among HIV infected patients in Addis Ababa, Ethiopia (n = 367).

Characteristic		Univariate Analysis				Multivariate Analysis			
		OR (95% CI)		P		OR (95% CI)		P	
Cryptococcal Antigen Positive		0.77 (0.22–2.65)		0.68		1.27 (0.33–4.83)		0.73	
Demographics									
Mean Age in years (IQR)		0.98 (0.94–1.01)		0.20					
Male		1.28 (0.68–2.41)		0.44					
BMI <18.5 kg/m^2^		1.71 (0.88–3.31)		0.11					
Site 1 vs. 2		1.23 (0.60–2.54)		0.57					
ART Use									
ART at Baseline		0.41 (0.21–0.78)		0.01					
ART at Follow Up		0.04 (0.02–0.09)		<.001		0.06 (0.02–0.19)		<.0001	
CD4 Count Status									
CD4<100 vs. 100–200 at Baseline		2.97 (1.56–5.63)		0.001		3.04 (1.37–6.73)		<.006	
Increase in CD4 count		0.08 (0.04–.17)		<.001		0.12 (0.05–.27)		<.0001	
Opportunistic Infections									
Treated for TB at Baseline or during FU	0.96 (0.36–2.59)		0.94						
Treated for Pneumonia at baseline or during FU	0.99 (0.37–2.67)		0.99						
Herpes Zoster at baseline or during FU	1.25 (0.46–3.41)		0.66						

BMI, body mass index; ART, antiretroviral therapy; TB, tuberculosis; FU, follow-up.

### Subset Analysis Among Persons with a CD4<100 Cells/µl

Outcomes were further analyzed among study individuals who had a CD <100 cells/µl at baseline. In the study, there were 117 persons with a CD4<100 cells/µl, including 13 who were serum CRAG positive. Poor outcomes were observed in 23% of those who were serum CRAG positive (3 of 13), as compared with 20% who were serum CRAG negative (21 of 104). 90% (94 of 104) of individuals with a CD4<100 cells/µl were on ART at baseline. Of the 13 patients with cryptococcal antigenemia, 10 were on ART at baseline including 2 of the 3 patients with poor outcomes. In univariate analysis, there was no association between serum cryptococcal antigenemia and a poor outcome among those who had a CD4<100 cells/µl at baseline (p = 0.81).

## Discussion

Our study found no association of cryptococcal antigenemia (aOR = 1.3, 95% CI 0.3–4.8) with a poor outcome (defined as mortality or lost to follow-up) at one year in a HIV-infected cohort in Addis Ababa, Ethiopia. In contrast to prior studies [7]–[9], our cohort included HIV-infected patients with CD4 counts <200 cells/µl who had a high rate of baseline ART use (74%). Our findings suggest that CRAG screening among ART-experienced patients would not improve clinical outcomes in patients with a CD4<200 cells/µl.

Our study is the first to investigate clinical outcomes of patients with cryptococcal antigenemia in an ART-experienced cohort. In particular, our study’s findings are different than those from previous studies among ART-naïve persons that found cryptococcal antigenemia to be predictive of the development of CM and a significant risk factor for mortality [7]–[9]. In studies from Uganda and South Africa, cryptococcal antigenemia was a strong predictor of mortality (HR = 4.3, 95% 1.5–12.1 and HR = 3.2, 95% CI 1.5–6.6, respectively). A major difference between these prior studies and our study is that we enrolled patients who had already initiated ART prior to CRAG screening while the prior studies enrolled ART naïve patients. Almost all cryptococcal antigenemic patients from our study were receiving ART (84%) at baseline and had been on for an average of 3 years [4]. Most unmasking of cryptococcal disease occurs in the first 4 months of ART initiation [7], [10], thus this ART-experienced population likely incorporates a survival bias. Our findings suggest that in ART-experienced patients with a CD4<200 cells/µl the presence of asymptomatic cryptococcal antigenemia does not negatively influence clinical outcomes.

Our results indicate that among ART-experienced persons, continued adherence to ART may be sufficient in controlling the progression of asymptomatic cryptococcal antigenemia in those with a CD4<200 cells/µl, except in the case of extremely high titers. In our cohort, poor outcomes occurred only among three patients with high CRAG titers (>1∶1024) while all CRAG positive patients alive at follow-up had a titer ≤1∶128. Notably all three individuals also had a CD4<100 cells/µl. A prior study from South Africa also found a correlation between poor outcomes and higher CRAG titers [7]. Moreover, the study also found that the augmentation of immune response from ART initiation in many cases resulted in effective clearance of asymptomatic infection. Among the antigen positive who did not develop cryptococcal meningitis, 78% had a fall in antigen titer similar in magnitude to that seen with effective antifungal treatment [7]. Our findings suggest that a very high CRAG titer portends a poor outcome even among ART-experienced patients and antifungal therapy should be initiated.

The results of our study emphasize that the initiation and continuation of ART is vital for improving survival and quality of life for persons living with HIV. The receipt of ART at last follow-up, an increasing CD4 count, and a CD4 count >100 were all associated with a reduced risk of a poor outcome in multivariate analysis. These findings are in accordance with the many studies demonstrating improved clinical outcomes and decreased mortality among individuals receiving ART in both developed countries [11]–[18] and RLS [19], [20]. Moreover, we observed lower rates of poor outcomes among individuals with serum cryptococcal antigenemia as compared to previous studies with ART-naïve individuals with a CD4<100 cells/µl [7], [8]. We conjecture that the low rate of mortality and development of cryptococcal meningitis observed among screen positive individuals in this cohort as compared to prior studies with ART-naïve individuals is related to high rates of successful ART receipt at baseline (26 of 31 of CRAG positive individuals), higher CD4 counts at baseline (18 of 31 had CD4>100 cells/µl), and a survivorship bias resulting from a long duration of previous therapy (37 months) [4].

Treatment guidelines for the management of cryptococcal disease were not followed in the two clinics studied, suggesting the need for continued improvement of HIV-related care. Infectious Diseases Society of America (IDSA) clinical practice guidelines recommend lumbar punctures and blood cultures to be performed on all patients with asymptomatic antigenemia, followed by treatment as symptomatic meningoencephalitis if positive. If there is no evidence of meningoencephalits, treatment with fluconazole is recommended until immune reconstitution has occurred. Immune reconstitution is defined as having a CD4 cell count **>**100 cells/µl, an undetectable or low HIV RNA level sustained for 3 months, and at least 1 year of antifungal drug exposure with close patient follow-up. Reinstitution of maintenance therapy is to be considered if CD4 decreases below 100 cells/µl [21]. Despite these recommendations, no patients in the two clinics had a lumbar puncture performed and only 3 (10%) of 31 received any antifungal therapy during follow-up. Our results call into question whether a lumbar puncture is necessary for CRAG positive patients who are receiving ART, are asymptomatic, and have no clinical suspicion of CM. Reasons for failure to treat with fluconazole requires further study.

Current WHO screening guidelines recommend serum cryptococcal antigen screening among ART-naïve individuals with a CD4<100 cells/µl [3]. In a subset analysis including only persons with a CD4<100 cells/µl at baseline, we found no association of serum cryptococcal antigenemia and a poor outcome in our ART-experienced cohort. In order to detect clinical significance (p<0.05, power of 80%), an estimated10,000 individuals would need to be included, assuming similar rates of antigenemia, receipt of ART, and poor outcomes.

A combined outcome of death and lost to follow-up was used to define a poor outcome as lost to follow-up has been found to be associated with poor outcomes in RLS. In a meta-analysis of one Indian and sixteen Sub-Saharan Africa studies, mortality among lost to follow-up patients was estimated to be 40% [22]. While this method can overestimate the rate of poor outcomes, its strength lies in its ability to capture all patients with true poor outcomes and to account for the lack of a system to track death in Ethiopia. Even when death alone was evaluated as a primary outcome, there was no difference among those with a positive or negative CRAG test.

Our study is subject to several limitations: neither lumbar punctures nor repeat CRAG testing were performed and the study was carried out at only two HIV clinics. A lack of lumbar punctures precluded us from evaluating rates of CM and whether lumbar punctures should be completed in asymptomatic cryptococcal antigenemic patients receiving ART. Serial CRAG testing would have allowed us to evaluate if receipt of ART and CD4 increase lead to clearance of cryptococcal antigenemia. Lastly, our study took place in only two sites in Addis Ababa and therefore care is needed before generalizing our findings.

## Conclusions

We found no association of cryptococcal antigenemia with a poor outcome (death or lost to follow-up) in an ART-experienced, asymptomatic cohort of HIV-infected persons in Ethiopia at one-year follow-up. Importantly, our study raises questions about the clinical significance of low-level cryptococcal antigenemia among patients receiving ART and supports interim WHO guidelines recommending CRAG screening only for ART-naïve patients with low CD4 counts as well as CSF testing in symptomatic patients presenting with meningitis.
